# Autoantibody to MDM2: A Potential Serological Marker of Systemic Lupus Erythematosus

**DOI:** 10.1155/2015/963568

**Published:** 2015-05-18

**Authors:** Yuan Liu, Liping Dai, Weihong Liu, Guixiu Shi, Jianying Zhang

**Affiliations:** ^1^Department of Rheumatology and Clinical Immunology, The First Affiliated Hospital, Xiamen University, No. 55, Zhenhai Road, Xiamen 361003, China; ^2^Department of Biological Sciences, The University of Texas at El Paso, El Paso, TX 79968, USA

## Abstract

*Introduction.* Systemic lupus erythematosus (SLE) is one of the systemic autoimmune diseases characterized by the polyclonal autoantibody production. The human homologue of the mouse double minute 2 (MDM2) is well known as the negative regulator of p53. MDM2 has been reported to be overexpressed in SLE animal model and to promote SLE. Since abnormally expressed proteins can induce autoimmune response, anti-MDM2 autoantibody was examined in SLE patients. *Methods.* Anti-MDM2 antibody in sera from 43 SLE patients and 69 healthy persons was investigated by ELISA. Positive samples were further confirmed by western blotting. The immunological feathers of anti-MDM2 positive sera were analyzed by indirect immunofluorescence assay. Anti-p53 was also investigated in SLE patients by ELISA, and the correlation of anti-MDM2 and anti-p53 was analyzed. *Results.* The presence of anti-MDM2 in SLE patients was 23.30%, much higher than normal healthy persons (4.30%). These anti-MDM2 positive sera present a nuclear staining pattern. The presence of anti-p53 in SLE patients was 39.50%, and the titer of anti-MDM2 was positively correlated with anti-p53 in SLE patients. *Conclusions.* Anti-MDM2 autoantibody was detected at high prevalence in SLE patients. The detection of anti-MDM2 in SLE patients should be clinically useful.

## 1. Introduction

Systemic lupus erythematosus (SLE) is one of the systemic autoimmune diseases characterized by the production of autoantibodies to cellular constituents [1]. Autoantibodies are widely used as biomarkers in many types of autoimmune diseases and other diseases such as cancer. One of the most important research areas in which autoantibodies are used is diseases diagnosis.

Besides its use in diagnosis, the detection of autoantibodies can also provide information about clinical manifestations or prognosis of some autoimmune diseases. The study on biological functions of autoantibody or its antigens can provide us with a better understanding of the mechanism of pathogenesis of autoimmune diseases and thus may give us new insights into the new strategies in autoimmune diseases treatment.

Several autoantibodies have been well characterized in SLE. Some autoantibodies are considered to be highly specific to SLE, such as anti-Sm and antiribosomal P. However, these autoantibodies are present in only about 15% and 10% of SLE patients, respectively [2, 3]. Though anti-dsDNA antibodies are found to be highly presented in SLE patients with prevalence of about 70%, its level fluctuates significantly according to disease activity and treatment [4]. Patients with SLE are still heterogeneous in clinical manifestations and serological characteristics. More new autoantibodies in SLE still need to be identified in order to further classify this disease or to better understand its pathogenesis.

The human homologue of the mouse double minute 2 (MDM2), also known as E3 ubiquitin-protein ligase, is known to degrade several central cell cycle regulators including p53 and retinoblastoma (Rb) protein which are involved in important processes such as cell apoptosis [5]. It was interesting that DNA viruses can specifically induce MDM2 expression and then cause B cell lymphoma [6]. This is a mechanism that might contribute in a similar manner to lymphoproliferation in SLE induced by self-DNA. It was further demonstrated that cytosolic DNA can trigger the expression and activation of MDM2. In MRL-Fas^lpr^ mice, an animal model of SLE, the expression level of MDM2 was found to be increased and to correlate with disease progression [7], which provides us with a new molecular target in SLE. Since abnormally expressed proteins can induce autoimmune response, overexpression of MDM2 in lupus may trigger the production of autoantibody which may serve as a new serologic marker in SLE.

In this study, we investigated the presence of autoantibody to MDM2 in sera of SLE patients and normal human sera (NHS). We found that autoantibody to MDM2 was highly presented in SLE patients, which may be used as a new serological marker or therapeutic target in SLE.

## 2. Materials and Methods

### 2.1. Sera and Patients

In the current study, 69 normal human sera (NHS) and 43 SLE patient sera were examined. These sera were obtained from the serum bank of Cancer Autoimmunity and Epidemiology Research Laboratory at University of Texas at El Paso (UTEP), which were originally provided by our clinical collaborators. The diagnosis of SLE was established according to the American College of Rheumatology criteria [8, 9]. The Institutional Review Board of UTEP and Collaborating Institutions has approved this study.

### 2.2. Expression and Purification of Recombinant MDM2 and p53

Recombinant protein of MDM2 and p53 was derived from our previous studies [10]. MDM2 and p53 cDNAs were subcloned into pET28a vector producing fusion proteins with NH-terminal 6x histidine and T7 epitope tags. Recombinant protein was further expressed in* E. coli* BL21 (DE3) and then purified using nickel column chromatography (Qiagen, Valencia, USA). Reactivities of the purified recombinant protein have been analyzed by electrophoresis on SDS-PAGE and determined with polyclonal anti-MDM2 antibody (GeneTex, Irvine, USA).

### 2.3. Enzyme-Linked Immunosorbent Assay (ELISA)

Standard protocol for ELISA was conducted as described in our previous study [11]. In brief, a 96-well microtiter plate was coated with recombinant MDM2 or p53 protein overnight at 4°C with a final concentration of 0.5 *μ*g/mL in phosphate-buffered saline (PBS). The antigen-coated wells were blocked with gelatin postcoating solution at room temperature for 2 h. Human sera were diluted at 1 : 100 and then incubated for 2 h at room temperature in the antigen-coated wells, followed by HRP-conjugated goat anti-human IgG. The substrate 2,2′-azino-bis-3-ethylbenzo-thiazoline-6-sulfonic acid (ABTS, Sigma-Aldrich, St. Louis, USA) was used as detecting reagent. The average optical density (OD) value at a wavelength of 405 nm was applied as data analysis. The cutoff value used to designate a positive sample was the mean OD value of 69 NHS + 2SD.

### 2.4. Western Blotting

Denatured recombinant MDM2 protein was electrophoresed on 10% SDS-PAGE and transferred to nitrocellulose membranes. After blocking in PBS with 5% nonfat milk and 0.05% Tween-20 for 1 h at room temperature, the nitrocellulose membrane was incubated overnight with 1 : 200 dilution of human sera at 4°C. HRP-conjugated goat anti-human IgG (Santa Cruz, USA) was then applied as secondary antibody at a 1 : 10,000 dilution. The immunoreactive bands were detected by ECL kit according to the manufacturer's instructions (Thermo Scientific, Waltham, USA).

### 2.5. Absorption of Antibodies with Recombinant Protein

The diluted SLE sera (1 : 80) were incubated with recombinant protein MDM2 (final concentration of recombinant proteins in the diluted human sera was 0.01 *μ*g/*μ*L) overnight at 4°C and then centrifuged at 10,000 ×g for 15 min. The supernatant was used for immunofluorescence assay.

### 2.6. Indirect Immunofluorescence Assay (IIFA)

Hep-2 antigen substrate for IIFA test system was incubated with diluted sera (1 : 80) and preabsorbed sera overnight at 4°C. FITC-conjugated goat anti-human IgG was then used as secondary antibody at a 1 : 100 dilution. Fluorescence microscope (Leica DM1000, Germany) was used for examination.

### 2.7. Statistical Analysis

All data were represented as mean ± standard deviation (SD). The frequency of autoantibody to MDM2 in the sera was compared using the *χ* ^2^ test with Fisher's exact test. Correlation coefficients were calculated using the Spearman rank correlation analysis. Statistical analysis was performed in SPSS13.0 software. *P* < 0.05 was considered statistically significant.

## 3. Results

### 3.1. The Prevalence of Autoantibody to MDM2 in SLE

**Figure 1:**
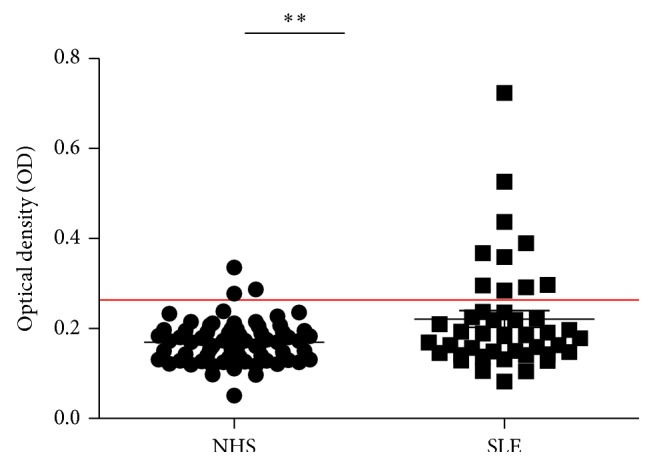
Titer of autoantibody against MDM2 in human sera by ELISA. The range of antibody titers to MDM2 was expressed as optical density (OD) obtained from ELISA. The mean + 2SD of NHS was shown in relationship to all serum samples. Titer of anti-MDM2 in SLE serum was much higher than that in NHS (*P* < 0.01). The cutoff value line for positive samples is indicated in the figure.

Serum level of autoantibody to MDM2 in SLE patients and normal human sera was determined by ELISA. The mean titer of autoantibody to MDM2 was significantly higher than that in NHS (Figure 1).

We then used the mean OD value plus 2SD of NHS as the cutoff value to determine the frequency of anti-MDM2 autoantibody positive sera in these three groups. The frequency of anti-MDM2 positive sera was significantly higher in SLE patients group (23.30%) than NHS group (4.30%) (Table 1).

**Figure 2:**
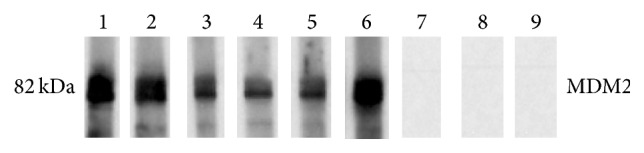
Western blotting analysis with representative sera in ELISA. Lanes 1–6: six representative SLE sera which were positive in ELISA and also had strong reactivity with MDM2 recombinant protein in western blotting analysis. Lanes 7–9: three randomly selected NHS had negative reactivity with MDM2 recombinant protein.

In order to confirm the presence of anti-MDM2 in SLE patients, anti-MDM2 autoantibody positive sera were further confirmed by western blotting. These sera also had strong reactivity with MDM2 recombinant protein in western blotting analysis (Figure 2).

### 3.2. Immunofluorescence Staining Pattern of MDM2 in Hep-2 Cells

**Figure 3:**
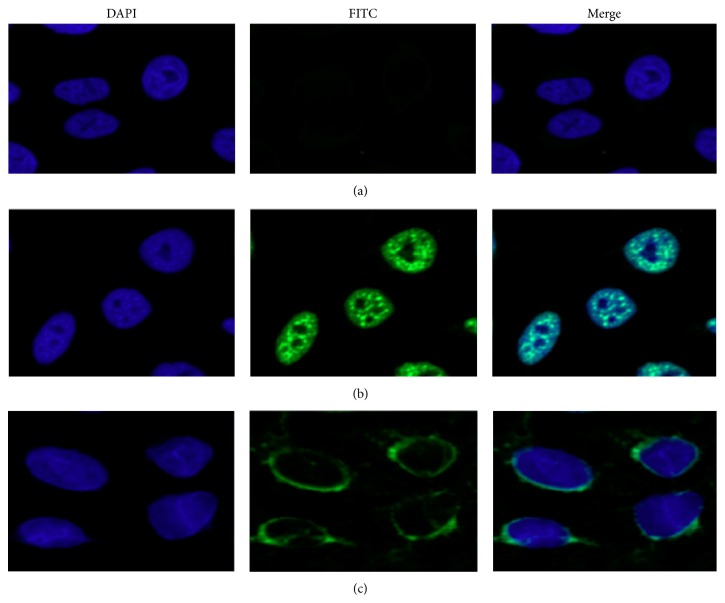
Representative immunofluorescence staining pattern from a SLE serum with anti-MDM2 autoantibody positive (performed on Hep-2 antinuclear antigen tissue slides). (a) NHS was used as negative control; (b) a representative SLE serum with anti-MDM2 autoantibody positive demonstrated an intense nuclear staining pattern; (c) the same SLE serum used in panel (b) was preabsorbed with recombinant MDM2, and the nuclear fluorescent staining was significantly reduced.

To further confirm the reactivity of autoantibodies against MDM2 in SLE sera and the intracellular localization of MDM2, commercially available Hep-2 cell slides were used in indirect immunofluorescence assay to examine anti-MDM2 autoantibody positive SLE sera. As shown in Figure 3, the anti-MDM2 positive sera had the nuclear staining patterns, while the normal human serum had very weak staining. The fluorescent staining was significantly reduced when the same serum was preabsorbed with recombinant anti-MDM2 protein.

### 3.3. Association of Anti-MDM2 and Anti-p53 in SLE Patients

Since MDM2 has been demonstrated as an important negative regulator of p53 and anti-p53 was also been reported to be found in SLE patients [12], whether there was an association between anti-MDM2 and anti-p53 is still unknown. We further investigated the presence of anti-p53 in these SLE patients by ELISA.

**Figure 4:**
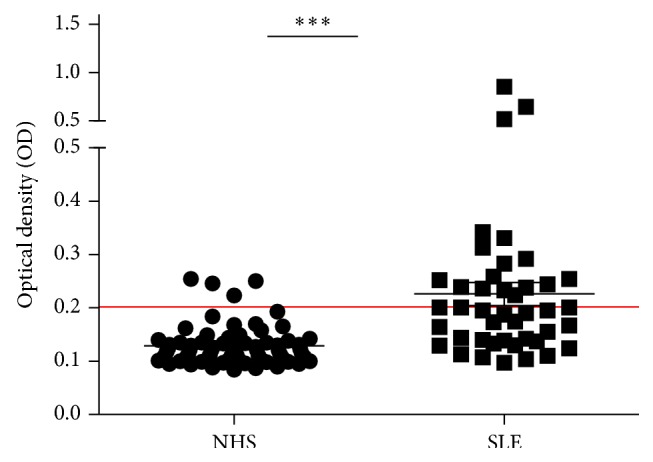
Titer of autoantibody against p53 in human sera by ELISA. The range of antibody titers to p53 was expressed as optical density (OD) obtained from ELISA. The mean + 2SD of NHS was shown in relationship to all serum samples. Titer of anti-p53 in SLE serum was much higher than that in NHS (*P* < 0.01). The cutoff value line for positive samples is indicated in the figure.

**Figure 5:**
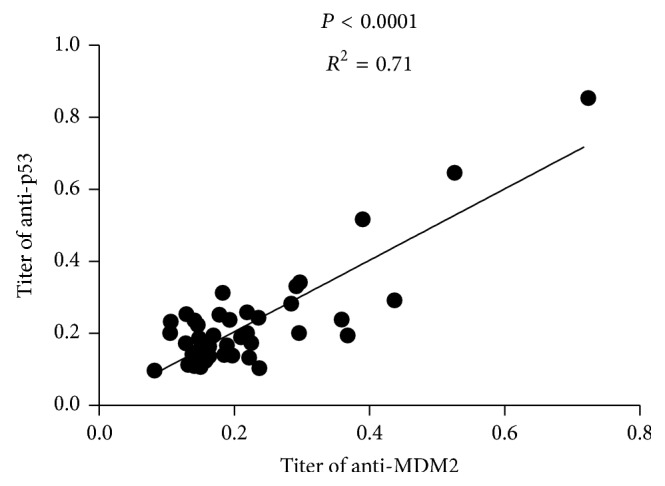
Correlation between anti-MDM2 and anti-p53 antibodies in SLE patients. A positive correlation between the titers of anti-MDM2 and anti-p53 antibodies was found in 43 SLE patient sera.

We used the mean OD value + 2SD of NHS as the cutoff value to determine the frequency of anti-p53 autoantibody positive sera. Consistent with results reported in other studies, the frequency of anti-p53 positive sera was significantly higher in SLE patients group (39.50%) than NHS group (5.90%) (Figure 4), and the titer of anti-MDM2 was positively correlated with anti-p53 (Figure 5).

## 4. Discussion

The present study showed that anti-MDM2 autoantibody was presented in 23.30% SLE patients, significantly higher than normal heathy humans. The titer of anti-MDM2 was positively associated with anti-p53. This suggests that the anti-MDM2 autoantibody might be used as a new serologic marker for SLE.

The MDM2 protein (also known in humans as Hdm2) was first identified as the product of a gene amplified over 50-fold on acentromeric extrachromosomal bodies (called “double minutes”) found in a 3T3DM spontaneously transformed mouse cell line [13, 14]. MDM2 was known as an important negative regulator of p53. Besides its regulation on p53, it was found that MDM2 also interacted with many proteins in addition to p53 such as NF-*κ*B [15, 16].

The role of MDM2 in cancer has been well studied. It was described as one of the tumor associated antigens (TAA) as MDM2 was overexpressed in several kinds of tumors [17]. It was demonstrated that MDM2 could elicit a functional autologous immune response in human [18]. It has also been reported that autoantibody to MDM2 can be found in patients with esophageal squamous cell carcinoma [19].

The role of MDM2 in immune regulation can be speculated by its regulation on p53. In recent years, p53 has been found to be important in both innate and acquired immune regulation [20, 21], and it was necessary in the inhibition of autoimmune inflammation [22]. Several studies have found that the presence of anti-p53 in SLE patient, with a prevalence of about 26%–59%, showed that anti-p53 was related to anti-DNA antibodies and can be used as a marker for detecting the disease activity of SLE [12, 23, 24]. Our study showed that anti-p53 was presented in 39.50% of SLE patients, and the titer of anti-p53 was positively correlated with anti-MDM2.

The direct role of MDM2 in immune regulation has been showed by several studies. Gasparini et al. showed that MDM2 can modulate dendritic cell-induced T cell proliferation [25] and Mulay et al. proved that MDM2 was required to induce mRNA expression and secretion of NF-*κ*B-dependent cytokines upon Toll-like receptor stimulation [26], which were important processes in SLE pathogenesis. Another study showed that MDM2 can promote SLE and inhibition of MDM2 can suppress the abnormal expansion of all T cell subsets, without causing myelosuppression effect on splenic regulatory T cells, neutrophils, dendritic cells, or monocytes [7]. These data suggest a new promising therapeutic target in SLE treatment. Our study further confirmed the importance of MDM2 in the pathogenesis of SLE and provided a new serological marker for SLE.

An increased risk of cancer in SLE patients has been observed [27, 28]. However, the mechanism underlying the association between SLE and cancer remained largely unknown. Whether the high prevalence of autoantibodies to MDM2 and p53 was related to the high risk of cancer in SLE patients still needs to be clarified.

Our study is the first one to demonstrate the presence of anti-MDM2 antibody in SLE patients. However, an obvious limitation of the present study is the limited clinical information of SLE patients included in this study. Analysis regarding the association of anti-MDM2 autoantibody and the clinical manifestations was hard to conduct due to the limited clinical information. Studies on anti-MDM2 in more SLE patients with detailed clinical information are needed to further ensure the role of anti-MDM2 in SLE diagnosis, disease activity evaluation, or prognosis prediction. The presence of anti-MDM2 still needs to be investigated in other autoimmune diseases such as rheumatoid arthritis, systemic sclerosis, Sjögren's syndrome, and dermatomyositis.

## 5. Conclusion

In conclusion, the present study reported a high prevalence of anti-MDM2 in SLE patients, which suggests the role of MDM2 in the pathogenesis of SLE. The detection of anti-MDM2 autoantibody may provide a new serological marker in SLE diagnosis.

## Figures and Tables

**Table 1:** Frequency of autoantibody against MDM2 in human sera by ELISA.

	Number	Anti-MDM2 (+)	Frequency
NHS	69	3	4.30%
SLE	43	10^*^	23.30%^*^

NHS: normal human sera; SLE: systemic lupus erythematosus.

^*^ *P* < 0.05.

## References

[1] Satoh M., Chan E. K. L., Sobel E. S. (2007). Clinical implication of autoantibodies in patients with systemic rheumatic diseases. *Expert Review of Clinical Immunology*.

[2] Tan E. M. (1989). Antinuclear antibodies: diagnostic markers for autoimmune diseases and probes for cell biology. *Advances in Immunology*.

[3] Yamasaki Y., Narain S., Yoshida H. (2007). Autoantibodies to RNA helicase A: a new serologic marker of early lupus. *Arthritis and Rheumatism*.

[4] Tan E. M., Smolen J. S., McDougal J. S. (1999). A critical evaluation of enzyme immunoassays for detection of antinuclear autoantibodies of defined specificities. I. Precision, sensitivity, and specificity. *Arthritis and Rheumatism*.

[5] Haupt Y., Maya R., Kazaz A., Oren M. (1997). Mdm2 promotes the rapid degradation of p53. *Nature*.

[6] Forte E., Luftig M. A. (2009). MDM2-dependent inhibition of p53 is required for epstein-barr virus B-cell growth transformation and infected-cell survival. *Journal of Virology*.

[7] Allam R., Sayyed S. G., Kulkarni O. P., Lichtnekert J., Anders H.-J. (2011). Mdm2 promotes systemic lupus erythematosus and lupus nephritis. *Journal of the American Society of Nephrology*.

[8] Tan E. M., Cohen A. S., Fries J. F. (1982). The 1982 revised criteria for the classification of systemic lupus erythrematosus. *Arthritis and Rheumatism*.

[9] Subcommittee for Scleroderma Criteria of the American Rheumatism Association Diagnostic and Therapeutic Criteria Committee (1980). Preliminary criteria for the classification of systemic sclerosis (scleroderma). *Arthritis and Rheumatism*.

[10] Zhang J.-Y., Megliorino R., Peng X.-X., Tan E. M., Chen Y., Chan E. K. L. (2007). Antibody detection using tumor-associated antigen mini-array in immunodiagnosing human hepatocellular carcinoma. *Journal of Hepatology*.

[11] Chen Y., Zhou Y., Qiu S. (2010). Autoantibodies to tumor-associated antigens combined with abnormal alpha-fetoprotein enhance immunodiagnosis of hepatocellular carcinoma. *Cancer Letters*.

[12] Aldar H., Lapa A. T., Bellini B. (2012). Prevalence and clinical significance of anti-ribosomal P antibody in childhood-onset systemic lupus erythematosus. *Lupus*.

[13] Cahilly-Snyder L., Yang-Feng T., Francke U., George D. L. (1987). Molecular analysis and chromosomal mapping of amplified genes isolated from a transformed mouse 3T3 cell line. *Somatic Cell and Molecular Genetics*.

[14] Fakharzadeh S. S., Trusko S. P., George D. L. (1991). Tumorigenic potential associated with enhanced expression of a gene that is amplified in a mouse tumor cell line. *The EMBO Journal*.

[15] Wade M., Wang Y. V., Wahl G. M. (2010). The p53 orchestra: Mdm2 and Mdmx set the tone. *Trends in Cell Biology*.

[16] Thomasova D., Mulay S. R., Bruns H., Anders H.-J. (2012). p53-independent roles of MDM2 in NF-*κ*B signaling: implications for cancer therapy, wound healing, and autoimmune diseases. *Neoplasia*.

[17] Wade M., Li Y.-C., Wahl G. M. (2013). MDM2, MDMX and p53 in oncogenesis and cancer therapy. *Nature Reviews Cancer*.

[18] Mayr C., Bund D., Schlee M. (2006). MDM2 is recognized as a tumor-associated antigen in chronic lymphocytic leukemia by CD8^+^ autologous T lymphocytes. *Experimental Hematology*.

[19] Chai Y. R., Peng B., Dai L. P., Qian W., Zhang Y., Zhang J. Y. (2014). Autoantibodies response to MDM2 and p53 in the immunodiagnosis of esophageal squamous cell carcinoma. *Scandinavian Journal of Immunology*.

[20] Šimelyte E., Rosengren S., Boyle D. L., Corr M., Green D. R., Firestein G. S. (2005). Regulation of arthritis by p53: critical role of adaptive immunity. *Arthritis & Rheumatism*.

[21] Menendez D., Shatz M., Resnick M. A. (2013). Interactions between the tumor suppressor p53 and immune responses. *Current Opinion in Oncology*.

[22] Zheng S.-J., Lamhamedi-Cherradi S.-E., Wang P., Xu L., Chen Y. H. (2005). Tumor suppressor p53 inhibits autoimmune inflammation and macrophage function. *Diabetes*.

[23] Herkel J., Mimran A., Erez N. (2001). Autoimmunity to the p53 protein is a feature of systemic lupus erythematosus (SLE) related to anti-DNA antibodies. *Journal of Autoimmunity*.

[24] Sherer Y., Gorstein A., Fritzler M. J., Shoenfeld Y. (2004). Autoantibody explosion in systemic lupus erythematosus: more than 100 different antibodies found in SLE patients. *Seminars in Arthritis and Rheumatism*.

[25] Gasparini C., Tommasini A., Zauli G. (2012). The MDM2 inhibitor Nutlin-3 modulates dendritic cell-induced T cell proliferation. *Human Immunology*.

[26] Mulay S. R., Thomasova D., Ryu M., Anders H.-J. (2012). MDM2 (murine double minute-2) links inflammation and tubular cell healing during acute kidney injury in mice. *Kidney International*.

[27] Bernatsky S., Ramsey-Goldman R., Clarke A. E. (2009). Malignancy in systemic lupus erythematosus: what have we learned?. *Best Practice & Research: Clinical Rheumatology*.

[28] Gayed M., Bernatsky S., Ramsey-Goldman R., Clarke A. E., Gordon C. (2009). Lupus and cancer. *Lupus*.

